# Vitamin D, infections and immunity

**DOI:** 10.1007/s11154-021-09679-5

**Published:** 2021-07-29

**Authors:** Aiten Ismailova, John H. White

**Affiliations:** 1 Departments of Physiology, McGill University, Montreal, Qc Canada; 2 Departments of Medicine, McGill University, Montreal, Qc Canada

**Keywords:** Vitamin D, Inflammation, Antibacterial, Antiviral, COVID-19, Cathelicidin, β-defensin 2, NOD2

## Abstract

Vitamin D, best known for its role in skeletal health, has emerged as a key regulator of innate immune responses to microbial threat. In immune cells such as macrophages, expression of CYP27B1, the 25-hydroxyvitamin D 1α-hydroxylase, is induced by immune-specific inputs, leading to local production of hormonal 1,25-dihydroxyvitamin D (1,25D) at sites of infection, which in turn directly induces the expression of genes encoding antimicrobial peptides. Vitamin D signaling is active upstream and downstream of pattern recognition receptors, which promote front-line innate immune responses. Moreover, 1,25D stimulates autophagy, which has emerged as a mechanism critical for control of intracellular pathogens such as *M. tuberculosis*. Strong laboratory and epidemiological evidence links vitamin D deficiency to increased rates of conditions such as dental caries, as well as inflammatory bowel diseases arising from dysregulation of innate immune handling intestinal flora. 1,25D is also active in signaling cascades that promote antiviral innate immunity; 1,25D-induced expression of the antimicrobial peptide CAMP/LL37, originally characterized for its antibacterial properties, is a key component of antiviral responses. Poor vitamin D status is associated with greater susceptibility to viral infections, including those of the respiratory tract. Although the severity of the COVID-19 pandemic has been alleviated in some areas by the arrival of vaccines, it remains important to identify therapeutic interventions that reduce disease severity and mortality, and accelerate recovery. This review outlines of our current knowledge of the mechanisms of action of vitamin D signaling in the innate immune system. It also provides an assessment of the therapeutic potential of vitamin D supplementation in infectious diseases, including an up-to-date analysis of the putative benefits of vitamin D supplementation in the ongoing COVID-19 crisis.

## Introduction

Vitamin D is obtained from few dietary sources such as oily fish and egg yolks, or from photochemical and thermal transformation of the cholesterol precursor 7-dehydrocholesterol in skin exposed to ultraviolet B radiation. However, the angle of the sun has to be greater than 45° for efficient synthesis of vitamin D to occur in skin. Consequently, it cannot be produced at latitudes of 45° or higher (which includes Canada and several European countries) during 6 months of the year or longer, a period known as vitamin D winter [1]. Vitamin D was discovered for its capacity to prevent rickets, a disease of bone growth that arises from insufficient uptake of dietary calcium [2]. Relevant to this review, a diagnosis of rickets is also associated with increased risk of development of diseases unrelated to calcium homeostasis, notably a number of immune disorders [3]. Unfortunately, vitamin D deficiency remains widespread; a study of adolescents in several European countries found that vitamin D levels in 80% of subjects were below the threshold of sufficiency. Moreover, over 40% were considered deficient or severely deficient [4]. This is consistent with estimates of dietary intakes in Europe, which were generally deficient in vitamin D [5], and the vitamin D status of the general European population, where vitamin D deficiency is widespread and most prevalent in dark-skinned subgroups [6]. Even in more southern countries, such as India, poor vitamin D status is common [7].

The vitamin D metabolite measured in most studies is the major form in serum, 25-hydroxyvitamin D (25D). 25D has a circulating half-life of several weeks. Consequently, its levels vary seasonally, and it represents a reliable indicator of vitamin D status [8]. 25D is generated by the mostly hepatic 25-hydroxylation of dietary or cutaneous vitamin D catalyzed by CYP2R1 and other enzymes [9, 10]. The active metabolite of vitamin D, 1,25-dihydroxyvitamin D (1,25D), is generated in peripheral tissues in a highly regulated manner by the unique 1α-hydroxylase CYP27B1 [11]. Originally CYP27B1 was considered to be found solely in the kidney, however this perception changed with evidence for its widespread presence in tissues unrelated to calcium homeostasis [11, 12]. 1,25D signals through the nuclear receptor vitamin D receptor (VDR), a ligand-regulated transcription factor [13]. Gene expression profiling studies have shown that 1,25D signaling directly and indirectly regulates over 1,000 genes in the human genome [14]. The most robustly induced 1,25D target gene is *CYP24A1*, which represents a negative feedback loop as it encodes the enzyme that 24-hydroxylates 25D and 1,25D to initiate their catabolism.

Importantly, the VDR and CYP27B1 are broadly expressed in many tissues that are not implicated in calcium homeostasis [11, 15]. Indeed, several non-classical, extra-skeletal actions have been attributed to vitamin D signaling. One of the most prominent of these is its role in regulating both the innate and adaptive arms of the immune system. This has gained added prominence over the past year or so in the wake of the COVID-19 pandemic, the disease caused by infection with the SARS-CoV-2 coronavirus, and a number of clinical trials have been conducted to investigate links between vitamin D and COVID-19. As detailed below, there is extensive and growing molecular evidence for a role of vitamin D signaling in the stimulation of innate immune responses to pathogen threat. Moreover, this is supported by an expanding body of clinical data. The goals of the present review are to explore the molecular basis for innate immune regulation by 1,25D, and the clinical evidence that vitamin D signaling promotes antibacterial and antiviral innate immunity, with a specific focus on COVID-19.

## 1,25D signaling contributes to innate immunity

**Fig. 1:**
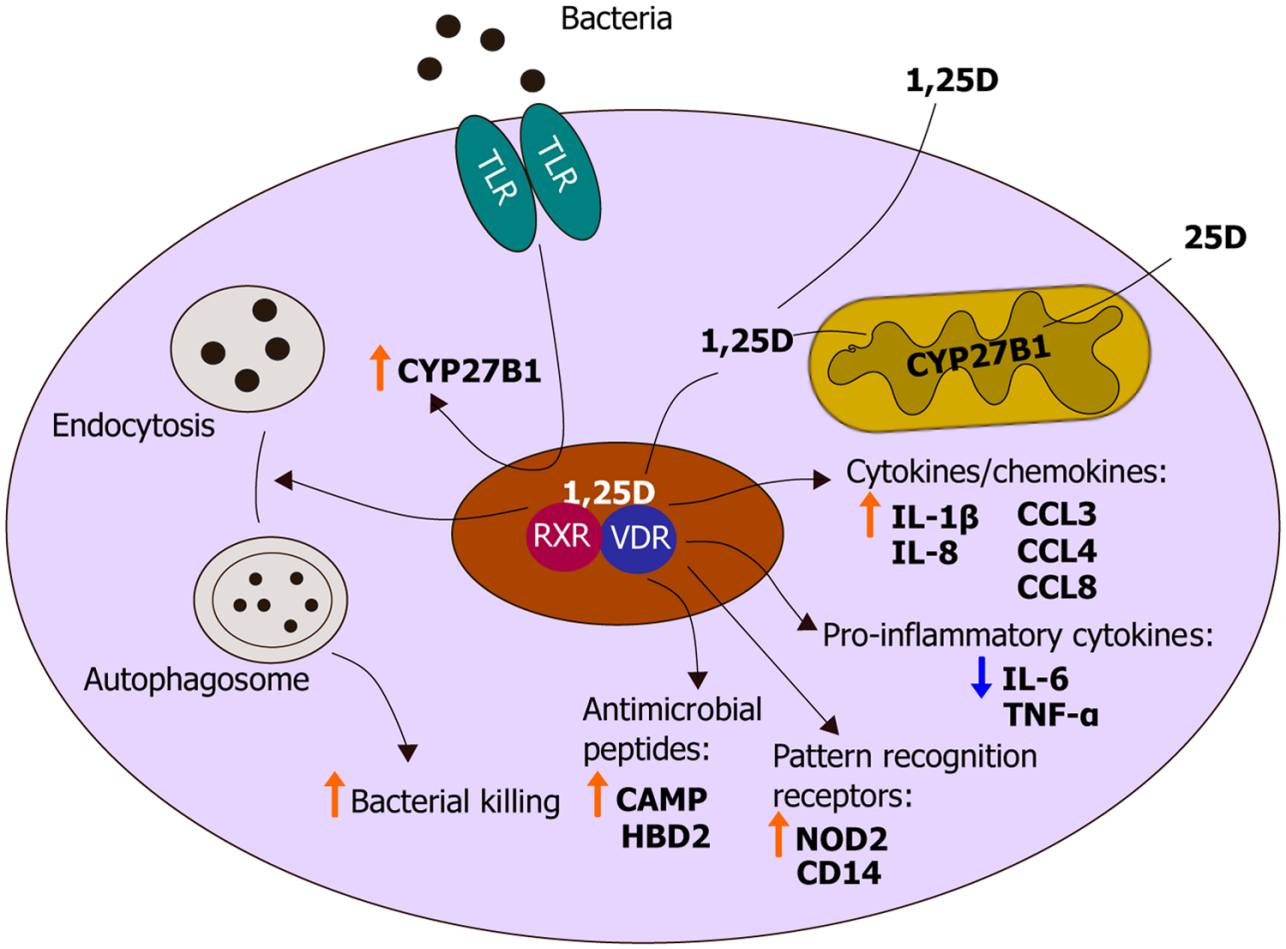
Antibacterial activity of vitamin D in monocytic cells. Schematic representation of the antimicrobial actions of vitamin D in response to bacterial infection. In monocytic cells, there is local production of 1,25D from circulating 25D, which is under control of pattern recognition receptor signaling. 1,25D bound to the VDR regulates expression of several genes essential for innate immune defense, including those encoding cytokines, chemokines, antimicrobial peptides and pattern recognition receptors. In addition, 1,25D suppresses production of pro-inflammatory cytokines, such as IL-6, preventing an overstimulated immune response. 1,25D can also induce autophagy, resulting in enhanced bacterial killing

Critical evidence for a physiological role of vitamin D signaling in immune system regulation came from observations that CYP27B1 expression in immune cells is controlled by a series of immune inputs (Fig. 1). The first compelling evidence for the importance of 1,25D signaling in innate immunity stems from a 2000 study by Mathieu and coworkers, who showed that murine *Cyp27b1* expression was inducible by bacterial lipopolysacharide (LPS) and interferon $\gamma$ (IFN-$\gamma$), a T cell cytokine released by pro-inflammatory Th1 cells [16]. In 2006, Modlin’s group exposed human macrophages *in vitro* to the ligand of the pattern recognition receptor toll-like receptor 2 (TLR2). This resulted in increased *VDR* and *CYP27B1* expression, as well as generation of endogenous 1,25D from 25D present in human serum [17]. Pattern recognition receptors (PRRs) are intracellular or transmembrane proteins that recognize microorganism-specific patterns or endogenous danger-associated molecules such as extracellular ATP, or cytoplasmic or endosomal nucleic acids [18]. As such, they are “first-responders” of the innate immune system as ligand binding to PRRs activate a cascade of signaling events that alert the cell of infection. *CYP27B1* transcription in macrophages and dendritic cells is also controlled by a cytokine network that includes IFN-γ [19, 20]. Taken together, these findings reveal that immune production of 1,25D is regulated by inputs that are unrelated to calcium homeostasis and that local synthesis of 1,25D and its downstream signaling are integral components of innate immune responses to microbes.

## Antibacterial responses to 1,25D

The last couple of decades of research have shown that locally produced 1,25D, via binding to the VDR, activates transcription of several genes associated with innate immunity. In human and murine cells, the gene encoding CD14, the co-receptor of the PRR TLR4 is strongly induced by 1,25D [21, 22] (Fig. 1). Furthermore, vitamin D signaling boosts expression of TLR2 in keratinocytes, and given that signaling via TLR2 or TLR4 ameliorates vitamin D signaling by increasing VDR and CYP27B1 expression, this constitutes a positive feedback loop [22]. In human macrophages, expression of the C-type lectin mannose receptor is repressed by 1,25D, which hampers infection by the dengue virus [23]. Convincing evidence for a direct regulation of antimicrobial innate immune responses by the hormone originated from finding vitamin D response elements (VDREs) adjacent to the transcription start sites of two genes that encode antimicrobial peptides (AMPs), cathelicidin antimicrobial peptide (CAMP/LL37) and β-defensin 2 (DEFB2/DEFB4/HBD2) [24]. A strong induction of *CAMP* by 1,25D was observed in several cell lines and human primary cell cultures, whereas 1,25D appeared to be a secondary inducer of *DEFB2*, notably enhancing interleukin 1β (IL-1β)-induced transcription of the gene [24] (Fig. 1).

Autophagy is an important facet of immune responses to intracellular infections and boosts microbial clearance [25, 26]. In macrophages, branched-chain amino acids (BCAAs), which are essential amino acids, act as nutrient sensors. Recently, we showed that 1,25D attenuated myeloid cell-specific, BCAA-dependent activation of the major metabolic kinase mammalian Target of Rapamycin (mTOR), which is a repressor of autophagy [27]. Autophagy induced by vitamin D signaling in macrophages (Fig. 1) was dependent on 1,25D-stimulated expression of branched-chain aminotransferase (BCAT1), the enzyme that initiates the catabolism of BCAAs; the effect of 1,25D on autophagy was eliminated in cells in which *BCAT1* had been ablated, thereby implicating enhanced BCAA catabolism in 1,25D-regulated autophagy in macrophages [27]. Collectively, the afore-mentioned results provide a mechanistic rationale for adequate vitamin D levels promoting widespread antimicrobial defense.

Mechanisms of immune signaling by vitamin D are not conserved among all species. The VDRE in the *CAMP* gene is present within an Alu repeat, a transposable element found uniquely in primates [28]; the transposition event seemed to have occurred in an early primate progenitor and, consequently, is evolutionarily conserved in Old and New World monkeys, apes and humans. There is also preservation of VDREs in *HBD2*, *CAMP*, and *NOD2* genes in primates, however, they do not appear to exist in rodents [29]. By the same token, transcription of the *HBD2*, *CAMP*, and *NOD2* genes is enhanced by 1,25D in human myeloid and epithelial cells, but not in murine cells [29]. Divergent mechanisms of AMP regulation in mice and humans may be due to the nocturnal nature of mice, which is contrary to daily human activity, enhancing the potential of vitamin D synthesis from the sun’s ultraviolet rays [17].

## Regulation of pattern recognition receptor and cytokine gene expression by 1,25D

Other than CD14 and TLR2, mentioned above, vitamin D can induce transcription of the intracellular pathogen sensing protein NOD2/CARD15/IBD1 in monocytes and epithelial cells [30] (Fig. 1). This PRR detects a lysosomal breakdown product of bacterial peptidoglycan known as muramyl dipeptide (MDP), and exposure to 1,25D and MDP initiates 1,25D and NF-κB-dependent induction of DEFB4 and CAMP in cells that express functional NOD2. In patients with the relapsing inflammatory bowel condition Crohn’s disease (CD) carrying inactivating *NOD2* mutations, this regulation was abolished [30]. Humans who are heterozygous or homozygous for inactivating mutations of the *NOD2* gene carry enhanced risks of developing CD of threefold and 20-fold, respectively [31]. Therefore, these findings indicate a link between 1,25D signaling and the pathogenesis of CD. 1,25D-regulation of NOD2 may also aid in combatting mycobacterial infections such as *M. tuberculosis* (*Mtb*); mycobacteria produce the N-glycolyl form of MDP, which binds to NOD2 with greater affinity than the N-acetyl form [32].

1,25D also directly induces transcription of the human gene encoding IL-1β, which is among the first cytokines generated in response to infection via pathogen-directed activation of the inflammasome [33]. A co-culture of *Mtb* infected macrophages with primary human airway epithelial cells revealed that 1,25D-mediated IL-1β secretion extended survival of infected macrophages (Fig. 1). Other cytokines/chemokines strongly upregulated by 1,25D in uninfected and *Mtb* infected macrophages include CCL3, CCL4 and CCL8, as well as the neutrophil chemoattractant, IL-8/CXCL8 [33] (Fig. 1). In *Mtb*-infected human peripheral blood mononuclear cells, dose-dependent suppression of the pro-inflammatory cytokines IL-6, TNF-α, and IFN-γ occurred with 1,25D treatment [34]; a VDR-initiated repression at the mRNA and protein level of the PRRs TLR2, TLR4, Dectin-1 and mannose receptor was postulated as the mechanism. Likewise, LPS-mediated production of pro-inflammatory cytokines IL-6 and TNF-α in human and rodent monocytes and macrophages was repressed by 1,25D [35, 36] (Fig. 1).

## Regulation by 1,25D in other innate immune cells

Most of the earlier work on vitamin D regulation in the innate immune system was performed in monocytes, macrophages and epithelial cells. Nevertheless, several different cell types express PRRs and possess the essential machinery to produce immune responses following microbial invasion. For instance, neutrophils express VDR mRNA to a similar extent to monocytes, and also induce expression of *CD14* and *CAMP* in the presence of 1,25D [24, 37]. Although they do not seem to express substantial levels of CYP27B1, they represent the major source of serum cathelicidin/LL37 encoded by *CAMP* due to their abundance in the blood [38]. Furthermore, mast cells also express VDR, but contrary to neutrophils, express CYP27B1 as well [39]. IgE-mediated mast-cell derived induction of proinflammatory and vasodilatory mediators in human and murine mast cells was shown to be inhibited by both 25 and 1,25D. Natural killer (NK) cells, which function at the frontier between innate and adaptive immunity, are also responsive to 1,25D signaling [40–42]. In patients with chronic renal failure and vitamin D-resistant rickets, there is dysregulated activity of NK cells, and supplementation with 1,25D is reported to ameliorate and normalize NK function in these patients [40, 43]. Moreover, in combination with the synthetic glucocorticoid dexamethasone, 1,25D enhanced expression of the anti-inflammatory cytokine, IL-10 in NK cells, inducing regulatory phenotype regulated by 1,25D signaling [42]. Globally, these findings demonstrate an important role for 1,25D innate immune regulation in a variety of immune cells.

## Evidence for regulation by 1,25D of antibacterial innate immunity *in vivo* in humans

Numerous clinical studies have provided evidence for 1,25D-mediated immune regulation. Pre-clinical data showed that 1,25D treatment enhanced secretion from cultured epithelial cells of antimicrobial activity against *Escherichia coli* and the lung pathogen, *Pseudomonas aeruginosa* [29]. Consistent with these findings, in a single-centre community-based randomised placebo-controlled double-blind trial, vitamin D supplementation (1000 international units per day for 90 days) led to more robust antimicrobial activity of lung airway surface liquid (ASL) [44]. Furthemore, increased ASL antimicrobial activity was observed in the summer-fall season compared to winter-spring, and an anti-LL37 (CAMP) antibody blocked the activity in ASL. In addition, vitamin D supplementation abrogated the seasonality of ASL antimicrobial activity [44]. A study conducted in Sweden reported an association between low vitamin D levels and an increased risk of dental caries. Strikingly, vitamin D status in participants correlated positively with levels of CAMP found in saliva [45]. Another study in Poland found similar results; investigators reported lower prevalence of caries in children supplemented with vitamin D [46]. Subsequently, it was found that CAMP/LL37 is efficacious against bacterial species that reside in plaque, such as *Streptococcus mutans* [47]. These findings provide a molecular basis for analyses of vitamin D supplementation trials. Vitamin D deficiency emerged as a risk factor for dental caries in a large-scale umbrella analysis of systematic reviews and meta-analyses of observational studies and randomized trials with vitamin D, which surveyed a total of 137 outcomes [48]. Similarly, a systematic review of controlled clinical trials concluded that vitamin D supplementation reduced rates of dental caries by about 50% [49].

Crohn’s disease arises from defective intestinal innate immunity, and vitamin D deficiency is common in patients with CD [50]. Several small clinical studies have been published providing evidence that vitamin D supplementation may be beneficial for patients with CD. These have recently been reanalyzed in two meta-analyses. From their survey of 18 randomized clinical trials, Li et al. found decreased relapse rates in CD patients treated with vitamin D relative to controls [51]. Similarily, a systematic review demonstrated low 25D status is associated with increased odds of inflammatory bowel disease (IBD) activity, mucosal inflammation, low quality of life and subsequent clinical relapse in CD patients [52]. These observations are likely to be reinforced in the future as additional findings continue to be published [53]. Recently, in a Korean cohort of IBD patients including those with CD, severe vitamin D deficiency was linked to an aggressive disease course and increased risk of surgical intervention [54]. These findings are of interest, because inactivating NOD2 mutations frequent in Western patients with CD are not found in affected Koreans (or in Japanese and Chinese CD patients) [55]. Other work in post-operative CD patients found that higher vitamin D levels were associated with lower risk of recurrence as measured by endoscopy [56]. Collectively, although it is not yet routine practice [57], these studies argue that vitamin D supplementation should be a part of a treatment plan for patients with CD.

## 25D signaling boosts antiviral immunity

Mechanistically, antiviral innate immunity is similar to its antibacterial counterpart. Specific PRRs sense viral pathogens and activate several downstream signaling events that initiate cytokine, type 1 interferon and antiviral effector responses [58, 59]. Intracellular or endosomal PRRs are stimulated by nucleic acids such as single-stranded RNA (ssRNA), and function as detectors of hijacking viral genomes. A restricted number of transcription factors, such as members of the interferon regulatory factor family, AP‐1 family members, and NF‐κB, initiate transcriptional responses to the recognition of viruses [60]. The initial transcriptional response and consequent interferon signaling result in the generation of antivral effectors.

**Fig. 2:**
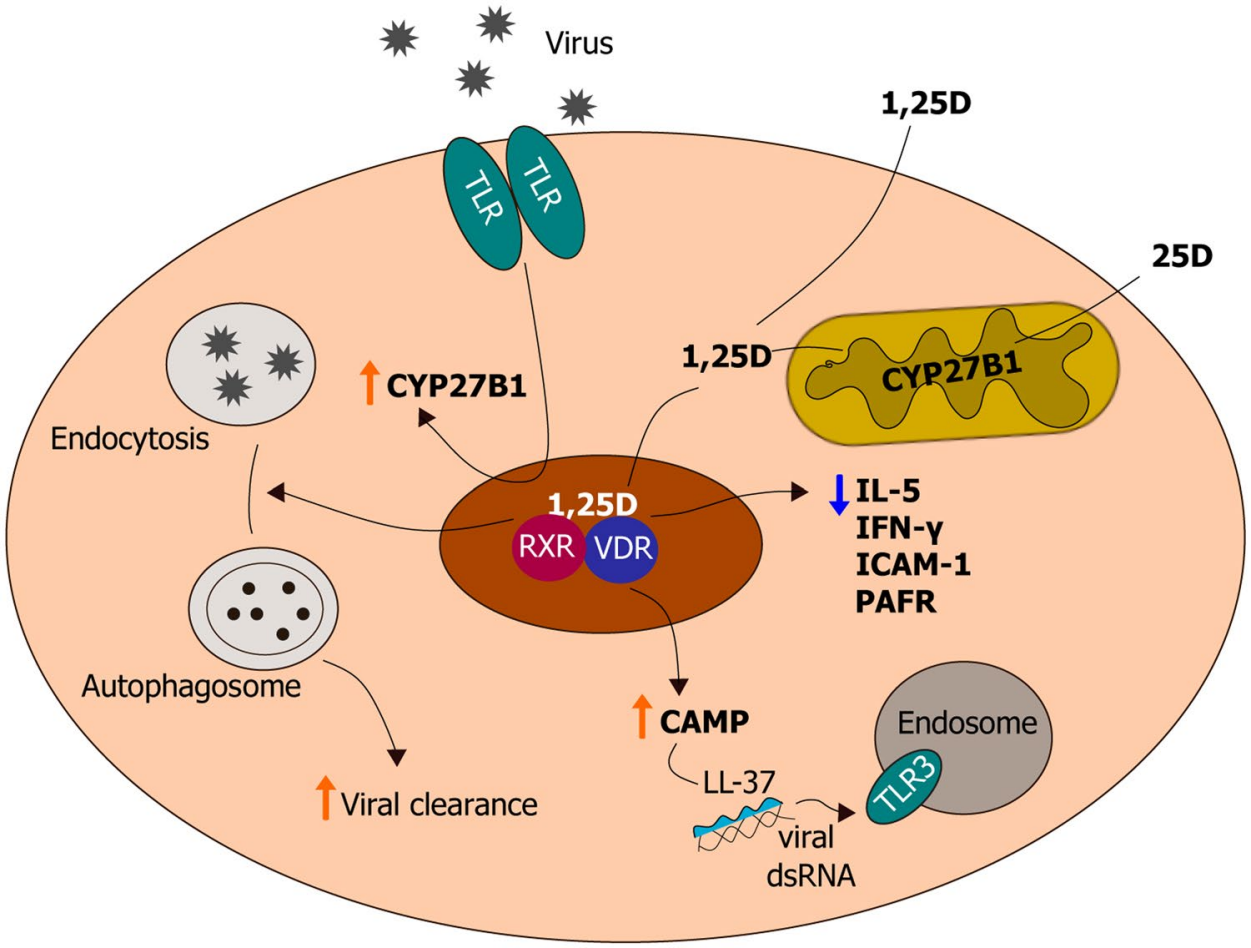
Antiviral activity of vitamin D in a lung epithelial cell. Illustration showing events underlying vitamin D metabolism and signaling during viral infection and responses induced by 1,25D. These include induction of CAMP expression as well as 1,25D-mediated suppression of inflammatory cytokines IL-5 and IFN-γ, ICAM-1, and PAFR. The mature form of CAMP, LL37, binds to viral dsRNA, which enables efficient binding to endosomal TLR3, augmenting TLR3 signaling and subsequent viral clearance. An additional mechanism for viral removal is the induction of autophagy by 1,25D

Clinical and epidemiological studies provide evidence for immune protection by vitamin D against a number of viruses, such as hepatitis viruses, human immunodeficiency virus, and viral respiratory pathogens [61]. Readers are referred to an extensive review by Lee et al. [62] of vitamin D signaling in anti-viral immunity for responses to non-respiratory viruses. Given that SARS-CoV-2, the etiological agent of COVID-19, primarily affects the lungs, we will consider vitamin D supplementation and viral respiratory tract infections in detail in this review. A meta‐analysis of epidemiological data suggests that supplementing with vitamin D daily or weekly decreased the incidence of acute respiratory tract infections, and that the effects were most striking in partipants with the lowest 25D status [63]. In addition, lung epithelial cells, which are responsive to 25 and 1,25D, are the target of most respiratory viruses. Primary human lung epithelial cells express CYP27B1, and, intriguingly, its expression was increased by dsRNA, which mimics viral genomes [64] (Fig. 2). Likewise, 1α‐hydroxylase activity is enhanced by respiratory syncytial virus (RSV) infection of bronchial epithelial cells [65]. Among the components of 1,25D-mediated antiviral immunity is the strong induction of *CAMP* gene transcription (Fig. 2). LL37, the active form of CAMP, binds to viral dsRNA, which facilitates its recognition by the PRR TLR3 [66] (Fig. 2). It has been suggested that this mechanism may be an outcome of the capacity of LL37 ability to promote wound healing, as augmented detection of extracellular nucleic acid from dying cells would expedite its clearance [67] (Fig. 2). Recently, LL37 was shown to bind directly to SARS-CoV-2 Spike protein and prevent binding to its receptor ACE2, which likely inhibits its viral entry into host cells [68]. Further, antimicrobial peptides that include LL37, are suggested to play a role in pulmonary defense against viruses by direct binding to and modulating cell-surface receptors, blocking viral replication and binding to virions [69].

Here, we will describe more in depth the molecular evidence for 1,25D-mediated protection against specific respiratory viruses. 1,25D may directly promote antiviral immunity against the influenza A (InA) virus, a highly contagious pathogen that leads to influenza outbreaks worldwide, mainly during the winter [70]. LL37 can bind directly to InA viruses and, according to electron microscopy data, the antimicrobial peptide can also disrupt viral membranes [71]. Likewise, the human and murine forms of CAMP decreased InA replication and disease severity in infected mice, in accordance with the above study’s findings [72]. Hayashi et al. [73] found that mice fed a diet consisting of a high dose of 25D and infected with the influenza virus exhibited decreased production of the inflammatory cytokines, IL-5 and IFN-γ (Fig. 2), as well as reduced virus replication and clinical outcomes of the infection compared to mice that were not supplemented with 25D. Furthermore, in InA-infected human A549 lung epithelial cells, 1,25D restored autophagic flux inhibited by the virus and thereby decrease apoptosis via a VDR-dependent mechanism [74] (Fig. 2). Infection with human RSV, one of the most common viruses to infect children and adults globally, can lead to upper and lower respiractory tract illness and in severe cases may progress to pneumonia, respiratory failure and death [75]. As mentioned above, RSV infection *in vitro* enhanced 1α‐hydroxylase expression (64). In turn, induction by 1,25D of antimicrobial peptide CAMP expression blocked RSV‐induced apoptosis and restricted viral replication i*n vitro* via suppressing assembly of viral particles [76]. One group found no significant effect of 1,25D was found on rhinovirus (RV) replication in primary human bronchial epithelial cells [77]. However, another study using primary cystic fibrosis bronchial cells, found that 1,25D dose-dependently suppressed RV load via induction of CAMP [78]. While RV infection decreased expression of the VDR and CYP24A1, treatment of infected cells with 1,25D reduced viral replication and release [65]. Greiller et al. found that treating A549 cells with 25D promoted transient resistance to RV infection [79]. This was attributed to suppression by 25D of RV-induced expression of intercellular adhesion molecule 1 (ICAM-1), a cell surface molecule that functions as the receptor for RVs, and platelet-activating factor receptor (PAFR) [79] (Fig. 2). Most recently, EPs®7630, a herbal drug preparation from the roots of *Pelargonium sidoides*, which is efficacious against respiratory tract infections, was found to increase host defense against RV by upregulating the VDR in human bronchial epithelial cells [80].

## Clinical evidence for vitamin D supplementation and COVID-19: results from a meta-analysis

Given the numerous accounts attributing an antiviral function for vitamin D signaling, it is appropriate to hypothesize that vitamin D may modulate the effects of the new severe acute respiratory syndrome coronavirus 2 (SARS‐CoV‐2) that has triggered the COVID-19 pandemic. This concept is elaborated in a number of comprehensive reviews [81, 82]. SARS‐CoV‐2 infection can be separated into three phases: (i) an asymptomatic phase with or without detectable virus; (ii) a non-critical phase with upper airway pathogenesis; and (iii) a severe, possibly fatal disease characterized by hypoxia, infiltration of ‘ground glass’ in the lung, and acute respiratory distress syndrome (ARDS), the principal cause of death in COVID-19 [83]. In general, coronaviruses enter cells via endocytosis, and subsequently release their viral RNA into the cytoplasm, produce viral proteins and initiate viral replication/transcription [84]. Although not fully determined, the most likely pattern recognition receptors involved in recognizing SARS-CoV-2 are TLR3 and TLR7 in the endosome, or the cytosolic sensors retinoic acid-inducible gene 1 (RIG-I) and melanoma differentiation-associated gene 5 (MDA5), which detect viral RNA [84]. These PRRs are directly linked by signaling cascades to initiate robust interferon responses [84], and an early peak of interferon signaling is followed by a decline in those responses in mild-to moderate COVID-19 [85]. However, levels of interferon responses are further increased in severely affected patients, suggesting that they are drivers of COVID pathology [85]. Moreover, numerous studies suggested that COVID-19 infection may be coupled with an inappropriate induction of immune responses which result in a “cytokine storm”, an unregulated response to systemic inflammation and secondary organ dysfunction as a consequence of pro-inflammatory cytokine and chemokine production by immune cells [84].

According to a current model, SARS-CoV-2 enters type II pneumocytes and macrophages via recognition of angiotensin converting enzyme 2 (ACE2) receptors, prompting quick virus entry and replication, as well as leading to a down-regulation of these receptors [86]. The reduction of ACE2 receptor activity induces activity of ACE1, thereby leading to the formation of angiotensin II [87]; high levels of angiotensin II may lead to ARDS or cardiopulmonary injury, which occur in severe COVID-19 cases [88]. Futhermore, viral infection results in the production of proinflammatory cytokines such as IL-1, IL-6, CXCL8, and TNF [85, 89, 90]. Increased production of these pro-inflammatory mediators results in an accumulation of inflammatory neutrophils and macrophages in the lungs, further inducing higher proinflammatory cytokine and chemokine production in bronchoalveolar lavage fluid and the circulation [84]. This cytokine storm was especially pronounced in critically ill patients, leading to ARDS, pulmonary edema, epithelial cell apoptosis and multiple organ exhaustion [91]. Futhermore, there is evidence that COVID-19 interferes with the cardiovascular system in hospitalized patients and is associated with conditions such as myocardial injury, acute coronary syndromes, arrthymias and thrombotic complications [82].

Epidemiological evidence and ecological studies suggest that vitamin D supplementation may be of therapeutic benefit in alleviating symptoms associated with COVID-19. For instance, there are several associations reported between COVID-19 and sun exposure [92]. An ecological study correlated low sun exposure, using proximity to the equator to estimate average exposure levels, with higher COVID-19 mortality in 88 countries [93]. This is further supported by observational studies regarding a higher number of deaths due to COVID-19 among individuals with darker skin [94, 95]. In addition, amid the elderly population, increased COVID-19 mortality was described to be linked with pre-existing, age-associated immune dysregulation coupled with vitamin D deficiency [96].

Mechanistically, 1,25D inhibits TNF/NFκB and IFN-γ signaling pathways [82], as well as decreasing the production of pro-inflammatory cytokines such as IL-6, which contribute to the cytokine storm phenomenon [97]. In addition, animal models of ARDS provide evidence that 1,25D can mitigate LPS‐initiated increased lung permeability by regulating activity of the renin–angiotensin–aldosterone (RAA) system and increasing expression of ACE2 [98]. This is supported by studies in *CYP27b1-/-* or *Vdr-/-* mouse models, which develop myocardial hypertrophies, with overexpression of components of the RAA system, hypertension and atherosclerosis [99, 100]. Moreover, vitamin D is a potent inhibitor of renin, a proteolytic enzyme that induces expression of angiotensin II, which as mentioned above, promotes increased COVID-19 severity [87]. The inhibition of renin by vitamin D was first demonstrated by a 2004 study, where vitamin D-deficient mice exhibited increased renin expression, whereas 1,25D injection suppressed renin synthesis in the mice [101]. The investigators also validated this finding in As4.1 cells, a murine renin-expressing cell line, in which 1,25D was found to directly attenuate renin gene transcription by a VDR-dependent mechanism [101]. Furthermore, cathelicidin, the antimicrobial peptide robustly induced by 1,25D, was shown to suppress hyperoxia-induced lung injury in newborn rats [102]. A number of meta-analyses of clinical studies support the preclinical data, as they link low 25D levels to heightened risk of cardiovascular outcomes, and ensuing mortality [48, 103–106]. COVID-19 is also associated with increased risk of coagulopathies and thrombosis [107]. In this regard, there are clinical reports linking poor vitamin D status to increased thrombotic events [108, 109]. A summary of the characteristics of COVID-19 pathogenesis and the effects of vitamin D is provided in Table 1.

**Table 1:** Summary of the symptoms contributing to COVID-19 pathogenesis and evidence for how vitamin D may be effective in combatting them

Features of COVID-19 pathogenesis	Effect of vitamin D on COVID-19
*1. Angiotensin II and the renin-angiotensin system*	
SARS-CoV-2 binds to ACE2 on alveolar cells [86] and impairs ratio of ACE2/ACE activity, which as a consequence, increases angiotensin II [88]	Increases ACE2 expression (98) and inhibits renin [101], both of which reduce angiotensin II levels
*2. Inflammatory cytokine storm*	
Increases production of pro-inflammatory cytokines leading to a cytokine storm [85, 89, 90]	Decreases production of pro-inflammatory cytokines (e.g. IL-6) that contribute to cytokine storm [97]
*3. Cardiopulmonary injury*	
Severe COVID-19 cases result in ARDS, pulmonary edema, cardiovascular injury, coagulopathies [82, 91], and thrombotic events [107]	Poor vitamin D status is associated with increased risk of cardiovascular outcomes and thrombosis [48, 103–106] 1,25D promotes production of cathelicidin, which can attenuate lung injury in a rat model of hyperoxia (102)

There is ample and growing clinical data exploring the association between vitamin D levels and COVID-19 pathogenesis. A meta-analysis by Kazemi and researchers surveyed 39 retrospective and prospective cohort, cross-sectional, case–control, and randomized controlled trial studies (up to November 26, 2020) to assess the relation between 25D status and SARS-CoV-2 infection as well as COVID-19 severity [110]. In reports that were adjusted and nonadjusted for confounders, the researchers found a greater risk of SARS-CoV-2 infection in the vitamin D deficient group. There were two compelling studies in this regard. An ecological study including 20 European countries concluded negative correlations between mean levels of vitamin D and COVID-19 infection and mortality rates [111]. Similarily, a cross-sectional study among 392 healthcare workers from the United Kingdom (UK) reported that vitamin D deficiency is an independent risk factor for COVID-19 seroconversion [112], a time period during which a specific antibody is detected in the blood in response to a virus [113]. Furthermore, in the studies that evaluated a link between vitamin D insufficiency and disease severity, most suggested such an association [110]. Among those reports were three randomized controlled trials (RCTs), the first of which was a pilot study performed by Castillo and co-workers at a university hospital in Cordoba, Spain [114]. Castillo et al. observed that only 1 out of 50 patients that supplemented with oral 25D required intensive care unit (ICU) admission, in comparison to the 13 out of 26 untreated counterpart that were admitted. While the results appear compelling, the study has been critized for being poorly controlled [114]. A subsequent RCT conducted in India validated the findings from the earlier Spanish study; high-dose oral vitamin D_3_ supplementation (60,000 IU per day for at least 7 days) in asymptomatic or mildly symptomatic SARS-CoV-2 RNA positive vitamin D deficient participants resulted in significantly enhanced SARS-CoV-2 viral clearance and decreased amounts of fibrinogen, an inflammatory marker associated with COVID-19 [115]. However, data from a multicenter, double-blind, randomized, placebo-controlled trial conducted in two centers in Sao Paulo, Brazil did not agree with the previous two trials [116]. One dose of 200,000 IU of vitamin D_3_ supplementation did not significantly attenuate length of hospital stay or other clinical outcomes, such as requirement for mechanical ventilation, compared with the placebo control group, as determined by the Brazilian RCT [116]. A limitation of this study, however, is that supplementation with vitamin D_3_ of their mostly obese population of patients (mean body mass index 31.6 kg/m2) may have been administered too late to considerably influence clinically relevant outcomes, as randomization occurred approximately 10 days following emergence of symptoms [117]. In addition, vitamin D_3_ supplementation is less efficacious at increasing serum 25D concentrations than 25D itself [117]. The final conclusion from Kazemi et al.’s meta-analysis, is that for studies that assessed a relationship between vitamin D insufficiency and ICU admission, pulmonary complications, hospitalization, and inflammation, results were inconsistent [110].

## Recent clinical data assessing an association between vitamin D and COVID-19 infection and course of disease

Since late 2020, there have been several studies published with the aim of determining whether supplementation with vitamin D can curb COVID-19 infection. A case–control study conducted over two months with 201 hospitalized patients and 201 matched controls from Iran described an inverse association between circulating 25D levels and COVID-19 infection [118]. Furthermore, in their single-center retrospective cohort study, Meltzer et al. found an increased risk for COVID-19 susceptibility among black individuals with 25D levels less than 40 ng/mL (100 nM) in comparison with those with 40 ng/mL (100 nM) or greater [119]. However, no significant associations were observed for white participants [119]. A retrospective case–control investigation in Saudi Arabia determined that, although low vitamin D levels may raise the risk for mortality, they are not linked to increased risk of SARS-CoV-2 infection [120]. Moreover, researchers at McGill University were concerned with vitamin D levels being associated with many confounding variables, such as age, sex and ethnicity, and hence there may not be a causal association with vitamin D and COVID-19 protection [121]. To circumvent this, the investigators obtained genetic variants from 443,734 individuals of European ancestry and used these for Mendelian randomization, a genetic tool to limit the confounding, to approximate the influence of increased vitamin D on COVID-19 outcomes. From this, they could not find evidence for a link between genetically estimated circulating 25D levels and COVID-19 infection, hospitalization, or severe disease. However, it is important to note that the combined effect of all variants that tend to lower 25D levels is only about 5% [121]. Further, a hospital-based cross-sectional investigation in India observed a prevalence of 59% and 89% of vitamin D deficiency and insufficiency, respectively, among 156 COVID-19 hospitalized patients [122].

An equally important goal is to determine vitamin D supplementation can prevent critical course of disease and death from infection by COVID-19. To this end, a large UK-based cross-sectional observational study recruited 986 COVID-19 patients from three different hospitals, 151 of who received 280,000 IU of vitamin D_3_, given either as separate weekly or daily doses over a time period of up to seven weeks [123]. This booster therapy of vitamin D led to a markedly decreased risk of COVID-19 mortality among those afflicted with the disease [123]. Consistent with this report, a cohort investigation of 30 ICU patients in Greece, as well as a correlation study from 157 residents of an Italian nursing home revealed reduced mortality from SARS-CoV-2 infection with vitamin D treatment [124, 125]. A retrospective chart review report conducted over five months at Boston University Medical Center also found similar results; an independent association between serum 25D 30 ng/mL (75 nM) or greater and reduced risk of mortality from COVID-19 in elderly and non-obese patients was described [126]. Additional support for this association is provided by an Italian prospective study, which noted significantly lower 25D levels in severely symptomatic COVID-19 patients, as well as those who died from the disease [127]. The investigators also found an inverse correlation between low 25D status and high levels of the pro-inflammatory cytokine, IL-6 [127]. Furthermore, 25D insufficiency was related to increased severity of lung consolidation, which occurs when air that fills the lungs is replaced with bodily liquid, longer duration of disease and risk of death in an elderly population with COVID-19 compared with age-matched control subjects [128]. Although 25D status was not correlated with changes in clinical course, low levels of 1,25D were linked with prolonged mechanical ventilation and a worse acute physiology and chronic health evaluation score in 26 patients receiving intensive care with confirmed SARS-CoV-2 infection and COVID-19 induced acute respiratory distress syndrome [129]. In a retrospective study conducted in Sicily, low levels of vitamin D were observed in 50 hospital patients with a positive polymerase chain reaction (PCR) for SARS-CoV-2 [130]. Despite this finding, an association could not be found between low vitamin D status and increased markers of inflammation or clinical severity [130]. In addition, a meta-analysis of 31 observational studies found a trend between serum 25D levels of less than 20 ng/ml (50 nM) and an increased risk of mortality, ICU admission and ventilation; however, these associations were not found to be statistically significant [131]. Nevertheless, another meta-analysis that included 47 countries from Europe and Asia, revealed a significant positive correlation between both COVID-19 infection and mortality rates and vitamin D deficiency [132].

## Ongoing trials of vitamin D and COVID-19

At the time of this writing, there are 54 ongoing clinical trials registered (www.clinicaltrials.gov) assessing links between vitamin D and COVID-19. Here, we will discuss a few of these current trials. VIVID (vitamin D for COVID-19) trial is cluster randomized, double-blinded trial that will test vitamin D_3_ supplementation for early treatment and post-exposure prevention of COVID-19 using 2700 participants in the US [133]. The proposed time frame of the study is 4 weeks and it is estimated to be completed on December 31, 2021 [133]. Another ambitious project is the CORONAVIT trial, a randomised clinical trial in the UK, to be completed by June 30, 2021, that will recruit 6200 residents 16 years of age or older to assess whether a “test-and-treat” approach to correct vitamin D deficient individuals will lead to a reduced risk and/or severity of COVID-19 [134]. Investigators will offer participants in the intervention group with 25D level < 30 ng/mL (75 nM) a daily low dose of 800 IU or a higher dose of 3200 IU vitamin D_3_, whereas those in the control group will receive the national recommendation of 400 IU per day of vitamin D for a period of 6 months [134]. Furthermore, the COVIDIOL trial in Cordoba, Spain follows the pilot study mentioned above with 76 participants [114], and will include an estimated 1008 patients aged 18–90 years with COVID-19 and a radiological image showing inflammatory pleuropulmonary exudate [135]. The intervention group will obtain the best available treatment plus oral 25D (0.532 mg = 21,280 IU on day 1, 0.266 mg = 10.640 IU on days 3, 7, 14, 21 and 28), whereas the control group is given only the best available treatment [135]. The trial will last 28 days in total and its end date is June 30, 2021 [135]. In addition, a high-single dose of vitamin D randomized double-blind clinical trial in Switzerland will be conducted with vitamin D-deficient COVID-19 patients aged 18 years or older [136]. The intervention group will receive a single oral high dose of 140 000 IU of vitamin D_3_ as well as continue with the standard dose of 800 IU daily, whereas the control counterpart will obtain one dose of placebo orally and also continue with the 800 IU dose per day. The study intends to recruit 80 patients and its estimated completion date is June 30, 2021 [136].

## Conclusions

There is growing evidence that vitamin D signaling is active throughout the immune system, and that it is physiologically important in protecting the human host from bacterial and viral invaders. Mechanisms of vitamin D-innate immune signaling include its production of cytokines, antimicrobial proteins, and pattern recognition receptors. Early laboratory studies showing that 1,25D stimulated antibacterial innate immunity are supported by increasing clinical evidence for the beneficial effects of vitamin D supplementation in bacterial infections. The actions of vitamin D in the immune system have also raised the possibility that vitamin D supplementation may combat viral infections, including those caused by SARS-CoV-2. There is evidence from several preclinical and clinical studies that vitamin D supplementation can attenuate viral respiratory tract infections. Vitamin D deficiency is common in most North American and European countries [137, 138], and is particularly prevalent in nursing homes [139, 140], which have been hard-hit by the COVID-19 pandemic. Many clinical reports suggest that vitamin D supplementation, at least for the elderly and patients with low 25D status, can help in protecting against COVID-19 infection and severe course of disease. However, globally, the clinical data for the beneficial therapeutic effects of vitamin D supplementation in COVID-19 is mixed, and it is clear that more research on the possible role of vitamin D in influencing the risk and course of COVID-19 disease is required.
